# Effects of Transcranial Direct Current Stimulation Combined with Physiotherapy on Gait Pattern, Balance, and Functionality in Stroke Patients. A Systematic Review

**DOI:** 10.3390/diagnostics11040656

**Published:** 2021-04-05

**Authors:** Víctor Navarro-López, Francisco Molina-Rueda, Samuel Jiménez-Jiménez, Isabel M Alguacil-Diego, María Carratalá-Tejada

**Affiliations:** 1 International Doctoral School, Faculty of Health Sciences, Rey Juan Carlos University, 28922 Madrid, Spain; v.navarro.fisioterapia@gmail.com; 2 Physical Therapy, Occupational Therapy, Rehabilitation and Physical Medicine Department, Faculty of Health Sciences, Rey Juan Carlos University, 28922 Madrid, Spain; francisco.molina@urjc.es (F.M.-R.); maria.carratala@urjc.es (M.C.-T.); 3 Texum Rehabilitation Center, 28821 Madrid, Spain; sjimeneztexum@gmail.com

**Keywords:** gait disorders, neurologic, physical therapy modalities, postural balance, stroke, transcranial direct current stimulation

## Abstract

Background: The effectiveness of transcranial direct current stimulation (tDCS) together with conventional physiotherapy in motor rehabilitation after stroke has been widely studied. Despite this, few studies have focused on its application in gait and balance rehabilitation. This review aimed to determine the efficacy of transcranial direct current stimulation combined with conventional physiotherapy on gait, balance, and the functionality of the lower limb after stroke. Methods: This review was conducted according to the Preferred Reporting Items for Systematic Reviews and Meta-Analyses (PRISMA) guidelines. Four electronic databases were systematically searched for relevant articles. Randomized clinical trials in English or Spanish that evaluated the use of the transcranial direct current stimulation, combined with physiotherapy, to improve gait, balance, and lower limb functionality after stroke were included. Main results: 10 articles were included, with a total of 222 subjects. Data about population, assessment tools, protocols, sessions, and results were extracted. The methodological quality of the included studies ranged between 3 and 5. Conclusion: The use of transcranial direct current stimulation combined with physiotherapy improves gait parameters, static and dynamic balance, and lower limb functionality in stroke patients. Long-term effects have not yet been demonstrated.

## 1. Introduction

Gait impairments are a common entity in stroke survivors. It is estimated that 50% of patients in the first phase of the pathology will not have the ability to walk; 20–40% will not be able to walk in the chronic phase of the disease (>6 months), and 11% will only be able to walk with the use of aids [1,2]. Gait patterns are heterogeneous in these subjects, with disturbances in different phases of the gait cycle, which differ from healthy controls, in addition to disturbances in temporospatial parameters even in those who walk independently [3,4,5]. To achieve an independent gait is one of the objectives of people who have suffered a stroke [6]. However, this does not only encompass the achievement of a household walk but also community ambulation (which oscillates between 0.66 and 1.31 m/s) [7], since this influences the quality of life perceived by the subjects at a psychological level [8]. Rehabilitation programs have been found to improve quality of life in this regard [9], so there have been many methods used in gait training. However, none of these have been proven to be superior to another [10]. Repetitive gait training is a method with great results in the early stages of stroke [11,12]. Examples of these therapies are bodyweight supported treadmill training (BWSTT) and robot assisted gait training (RAGt) [12]. According to the most current therapies, noninvasive brain stimulation (NIBS) has shown promising results in the treatment of stroke survivors [13]. One of the most widely used methods is transcranial direct current stimulation (tDCS) [14]. According to several studies, tDCS can promote motor recovery in stroke patients by modifying cortical excitability (it appears to modify the discharge threshold of cortical neurons, using low-amplitude currents (0.5–2.0 milliamps or mA)) [15]. The way in which tDCS modulates cortical excitability is polarity-dependent, with several application models: (a) anodic stimulation, which increases cortical excitability by decreasing the excitability threshold; this model in stroke is placed in the injured cerebral hemisphere, (b) cathodic stimulation, which decreases cortical excitability by increasing the excitability threshold; this model in stroke is placed in the cerebral hemisphere contralateral to the stroke, (c) dual stimulation, which is a combination of anodic stimulation on the affected cerebral hemisphere and cathodic stimulation on the non-affected [16,17].

It is important to consider that tDCS is a technique with great inter-individual variability between subjects [15]. Even so, its efficacy in functional lower limb recovery remains unclear, and studies are limited [18]; those that do exist claim that more careful protocols are necessary due to the lower limb motor cortex representation proximity of both hemispheres and may produce the same sign modulation; in which both hemispheres would be stimulated with the same type of current, without producing the wanted therapeutic effects [19]. In addition, 2 milliamps (mA) stimulations will be necessary to achieve activity in the cortical representation of the lower limbs since these are located in the interhemispheric fissure, at a greater depth in the cerebral cortex compared to the cortical representation of the upper limbs, which will need lower intensity stimuli to be excited (1–1.5 mA) [20].

Although there are not many studies that support the use of tDCS in gait, there are several investigations focused on demonstrating its effectiveness in both gait training and lower limb rehabilitation, especially in combination with other therapies. Some recent systematic reviews and meta-analyses [21,22,23] studied the effectiveness of tDCS in the rehabilitation of gait and lower limb functionality. Other reviews [24] focused on the efficacy of tDCS in balance rehabilitation. These reviews analyzed articles that combined the tDCS with conventional physical therapy (PT) or robotic gait training. In this sense, the objective of the present study was to determine the efficacy of the tDCS combined with conventional PT on gait, balance, and lower limb functionality after stroke due to the limitations of some clinicians in the application of instrumental PT methods.

## 2. Materials and Methods

### 2.1. Clinic Question

The clinic question was posed according to the “population, intervention, comparison, and results” (PICO) model. Studies were included if they involved patients diagnosed with stroke who underwent an intervention using tDCS, combined with conventional PT, focused on the rehabilitation of locomotor gait patterns and balance, comparing its application to conventional PT alone. Conventional therapy was understood as any intervention of PT that did not use an instrumental process. The outcomes found in these studies had to be evaluated by quantitative or qualitative methods that measured gait coordination, gait spatial-temporal parameters, balance, functionality, and quality of life. 

### 2.2. Search Strategy and Database

The following databases were searched in March 2020: MEDLINE Complete, PubMed, Scopus, and SciELO. Some medical subject headings, such as “Stroke”, “Transcranial Direct Current Stimulation”, “Gait Disorders, Neurologic”, “Postural Balance”, or “Physical Therapy Modalities”, were used along with other related terms and were combined with the Boolean operators AND and OR. For the present review, experts were also consulted; the bibliography of the reviewed studies, gray literature, in addition to searching for protocols published in the registries, was reviewed for the inclusion of possible studies. Medline complete search is summarized in Table 1.

### 2.3. Eligibility Criteria

We included randomized clinical trials that investigated the effects of tDCS on gait and balance of stroke patients. We included studies with at least one group treated with tDCS combined with conventional PT and one control group treated with conventional PT treatment with sham tDCS or without tDCS. We included studies that applied any tDCS protocol, with intensities from 0.5 to 2 mA. Studies had to include measures of gait coordination, gait speed, or balance. If the studies showed measures of lower limb functionality, lower limb strength, and risk of falls, that would be included as secondary outcomes. Finally, only articles in English or Spanish were included, without limiting the year published.

### 2.4. Study Selection and Data Extraction

The clinical question was identified according to the PICO format, extracting the population included in the study (n), the inclusion criteria applied, the measures used for the participant evaluation, the description of the intervention carried out, and the results obtained. Titles and abstracts were screened by two independent authors to select which studies met the inclusion criteria; disagreements related to the eligibility criteria were solved by a third researcher. For the potential studies that met the inclusion criteria, full text articles were obtained. A new screening was performed independently by two researchers to choose the studies included in the review.

### 2.5. Methodological Quality Assessment and Risk of Bias

To analyze their methodological quality and guarantee their objectivity, the Oxford quality scoring system (Oxford, UK, Jadad, 1996) [25] was used. We divided the articles into three scores: (<3) indicated poor methodological quality, (3–4) indicated a moderate methodological quality, and (5) indicated a high methodological quality.

To assess the risk of bias of each randomized clinical trial, the Cochrane library criteria were used, based on the criteria of random sequence generation, allocation concealment, blinding of participants and personnel, blinding of outcome assessment, incomplete outcome data, selective reporting, and other bias. Study data were scored according to three criteria: “Low risk”, “High risk”, or “Unclear risk”.

The review was carried out following the PRISMA statement (checklist for systematic reviews and meta-analysis) [26]. Both methodological processes were carried out by two independent researchers; in case of different scores, a third researcher resolved the differences.

## 3. Results

### 3.1. Study Selection

**Figure 1:**
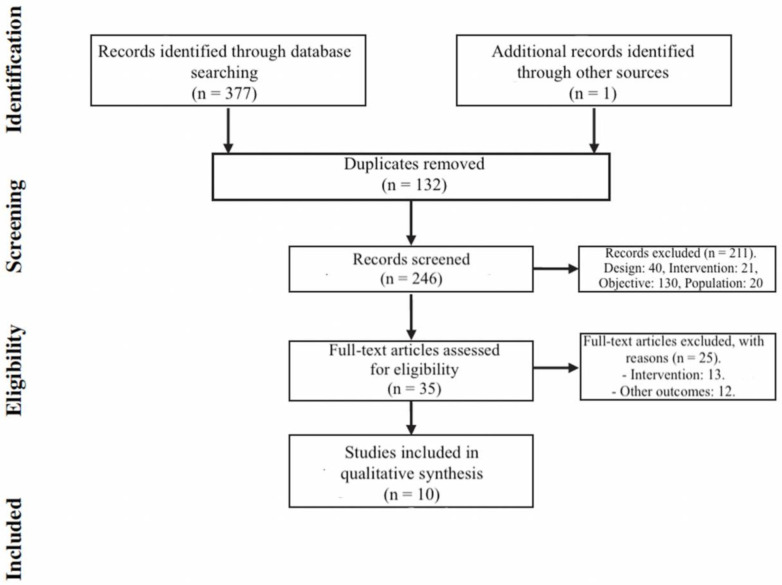
Flowchart of the selection process.

A total of 377 articles were found, duplicated studies were eliminated, leaving a total of 245 original articles. A standard screening checklist based on the eligibility criteria was used; studies other than randomized clinical trials in a non-relevant population that had a different intervention than tDCS or with a different objective on gait were not selected. The abstracts of the potentially relevant articles were read to see if they answered the research question and met the inclusion criteria. Ten studies were included in this review [27,28,29,30,31,32,33,34,35,36], with a total of 222 subjects. The selection process is shown in the flowchart (Figure 1). The 25 excluded articles and their reason for exclusion are shown in the Appendix A.

### 3.2. Study Characteristics and Result Synthesis

According to the primary outcomes, six studies evaluated gait patterns and temporal-spatial parameters, three studies used three-dimensional systems, one study used the Rivermead Motor Assessment (RMA), two studies used the functional ambulatory category (FAC), one study used the 10-m walking test (10MWT), and three studies used the 6-min walking test (6MWT). Nine studies evaluated balance, four studies used the timed up-and-go test (TUG), two studies used the Tinetti assessment tool (POMA), two studies used the Berg Balance Scale (BBS), one study used posturography, and one study used balance platforms.

According to the secondary outcomes, two studies evaluated functionality, one used the Fugl–Meyer Assessment lower limb sub-scale (FMA-LE), one used the Rivermead Mobility Index (RMI), three studies evaluated lower limb strength, two used dynamometry, one study used electromyography, six studies evaluated the risk of falls, one study used the rate of falls (RF), two studies used the Tinetti assessment tool, and four studies used the TUG.

The number of sessions ranged from 1 to 16. Five studies evaluated a single treatment session [30,31,34,35,36], of which three showed significant improvements in terms of gait, balance, and strength [31,34,35], and two showed no significant differences [30,36]. Five studies evaluated the results of several treatment sessions [27,28,29,32,33], of which three showed significant improvements in terms of gait, functionality, balance, and RF [27,28,32], and two showed no significant differences [29,33].

Regarding tDCS modalities, there were three different montages: (a) cathode tDCS [27,29], (b) anode tDCS [27,28,31,32,34,36], and (c) dual-tDCS (anode + cathode) [27,30,33,35]. Among the studies that applied cathodic stimulation, Andrade et al. [27] showed significant improvements in gait, measured by 6MWT and balance, measured by BBS and RF, while Fusco et al. [29] found no significant improvements.

About anodic stimulation, three studies found improvements in gait [27,31,32], two studies found improvements in balance [27,34], and two studies [28,36] did not find improvements in balance or gait. Andrade et al. [27] showed significant improvements in gait measured by 6MWT and balance measured by BBS and RF. Sohn et al. [34] showed significant improvements in static balance measured with a stabilometric platform and knee extensors strength measured with computerized robotic dynamometry. Ojardias et al. [31] showed significant improvements in terms of gait measured with the 6MWT. Finally, Park et al. [32] showed significant improvements in gait speed and temporospatial gait parameters measured by GAITRite (CIR Systems, Inc, Franklin, NJ/USA).

Four studies assessed the application of dual tDCS [27,30,33,35]. Three studies found significant improvement in gait and balance [27,33,35], and one study found no significant improvement [30]. Andrade et al. [27] showed significant improvements in gait measured by 6MWT and balance measured by BBS and RF. Saeys et al. [33] showed significant improvements in balance measured by POMA and in the lower limb functionality measured by RMA, and Tahtis et al. [35] showed significant improvements in dynamic balance measured with the TUG.

Concerning the stroke phase of the participants recruited, there were seven studies based on acute and sub-acute stroke subjects [27,28,29,30,33,34,35] and four studies based on chronic stroke patients [29,31,32,36]. On the subject of the studies that evaluated individuals in the acute/subacute stages, four studies found significant improvements in terms of gait and balance [27,28,34,35], and three studies did not show significant differences between the groups [29,30,33]. Long-term efficacy was measured by two studies [27,29]. Among them, the study carried out by Andrade et al. [27] showed significant improvements at 1 to 3 months of follow-up.

The characteristics of the studies included are summarized in Table 2. The included studies enrolled acute/sub-acute (<6 months (*n* = 170)) and chronic (>6 months (*n* = 52)) stroke; according to the protocol, four studies (*n* = 124) [27,30,33,35] used dual tDCS (anodal + cathodal), six studies (*n* = 147) [27,28,31,32,34,36] used anodal tDCS and two studies used cathodal tDCS (*n* = 71) [27,29]. The intensities ranged from 1.5 mA in two studies [29,33] to 2 mA in eight studies [27,28,30,31,32,34,35,36]. The duration of the sessions was 10 min in four studies [28,29,34,36], 15 min in two studies [32,35], 20 min in three studies [30,31,33], and one study did not specify the duration [27]. The number of sessions was 1 in five studies [30,31,34,35,36], 10 in three studies [27,28,29], 12 in one study [32], and 16 in one study [33].

### 3.3. Quality Assessment

We applied the Oxford quality scoring system to all studies, obtaining seven studies with a moderate methodological quality (3–4/5) [28,30,31,32,34,35,36] and three studies with high methodological quality (5/5) [27,29,33]. All studies had blinded participants, and 7 of 10 were double-blind.

### 3.4. Risk of Bias

**Figure 2:**
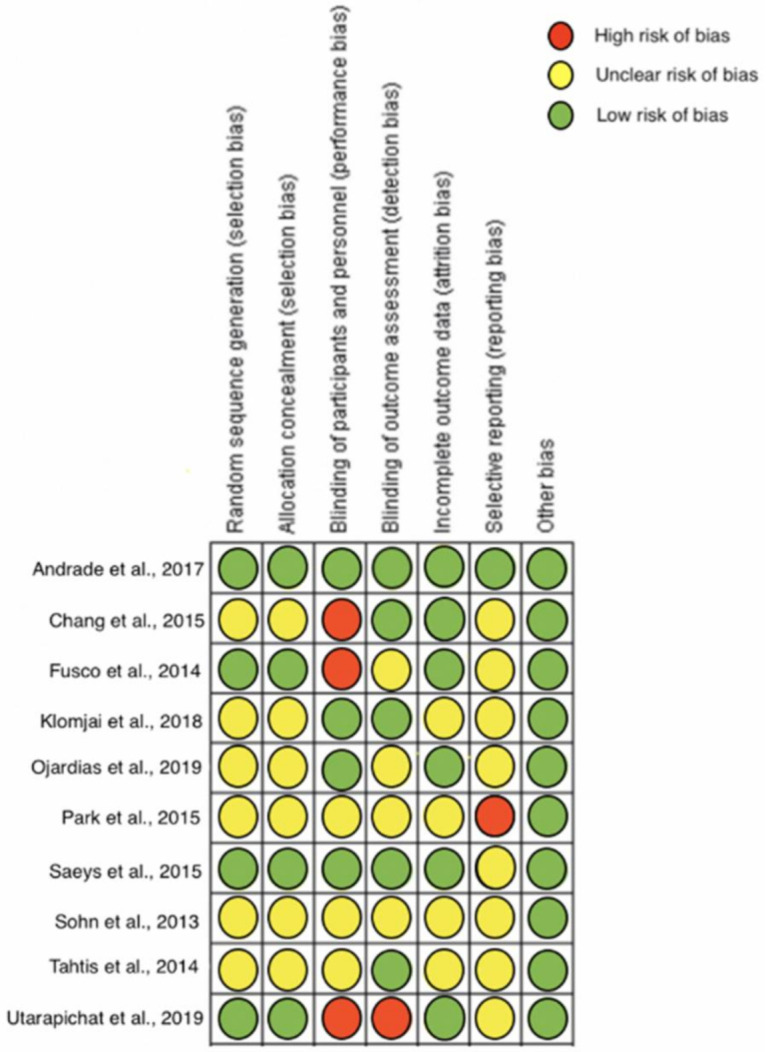
Risk of bias of the included studies.

Six of the included studies showed an unclear risk [28,30,31,32,34,35] and four studies showed a low risk [27,29,33,36] for selection bias. Regarding the performance bias, three studies showed a high risk [28,29,36], three an unclear risk [32,34,35], and four a low risk [27,30,31,33] in the blinding of participants, with one study showing a high risk [36], four studies unclear risk [29,31,32,34], and five studies low risk [27,28,30,33,35] in the blinding of outcome assessment. Regarding the attrition bias, four studies showed an unclear risk [30,32,33,35] and six studies a low risk [27,28,29,31,33,36] for the incomplete outcome bias, with one study showing a high risk [32], eight an unclear risk [28,29,30,31,33,35,36] and one [27] a low risk for selective reporting bias. No studies were considered to have any other type of bias (Figure 2).

## 4. Discussion

Similar studies in stroke patients were grouped in which the same parameters were compared whenever possible. Regarding the risk of bias, the item with the highest risk of bias was the blinding of participants and staff. Moreover, we found three articles with a high risk due to not blinding the therapists.

The sessions lasted between 10 and 20 min, according to what was established by Jeffery et al. [20]. The authors suggested that to achieve excitation in the lower limb motor cortex areas, 2 mA intensities are required for at least 10 min, these intensities being higher than those used to stimulate the upper limb motor cortex (1 mA). The stimulation intensities in the included studies were between 1.5 and 2 mA. In the studies that worked with intensities lower than 2 mA [29,33], no significant differences were observed between groups. It seems that currents of 2 mA should be used to achieve significant improvements in applications aimed at improving parameters related to the lower limb, such as gait, balance, risk of falls, or functionality and strength of the lower limbs.

The number of sessions ranged from 1 to 16, leading us to establish two groups: short-term and long-term studies. Similar improvements were found in both groups. The studies carried out by Ojardias et al. [31], Sohn et al. [34], and Tahtis et al. [35] found significant improvements in terms of balance, static balance (eyes closed and opened), quadriceps isometric strength, and gait after one tDCS session. Furthermore, Madhavan et al. [37] measured the precision of ankle movements following visual feedback with the foot, finding that the group that received a single session of tDCS improved more rapidly than the group that received sham stimulation (5 min vs. 10 min). Similar results were also found by Kaski et al. [38] in leukoaraiosis subjects after one tDCS session, where the authors observed improvements in gait velocity, stride length, stride length variability, and dynamic balance, assessed by video analysis and the TUG test. Five studies evaluated the results of several treatment sessions [27,28,29,32,33]. These included the studies carried out by Andrade et al. [27], Chang et al. [28], and Park et al. [32], who found significant improvements in terms of gait parameters, lower limb functionality, balance, and RF. The studies that did not find improvements were those that worked with lower intensities than 2 mA. Among these studies, two followed the sample for 1 to 3 months [27,29], finding maintenance of improvements at three months follow-up in the study carried out by Andrade et al. [27]. However, the studies carried out by Danzl et al. [39] and Geroin et al. [40], both using RAGt, did not find significant improvements between the study groups. The study of Geroin et al. [40] worked with intensities and times lower than those recommended. In contrast, the study of Seo et al. [41] found improvements in the FAC test and the 6MWT in 21 subjects with chronic stroke treated by RAGt. Although a single session and several treatment sessions have shown statistically significant improvements in favor of tDCS, the difference between the applications could be the duration of the improvements. Single sessions of tDCS have modified cortical excitability for a few minutes, while several treatment sessions have modified cortical excitability for hours, even a day [42]. This is clinically relevant for use in combination with physical therapy, as a long-lasting state of increased (or decreased) excitability will be vital to improve the neuroplastic brain changes.

Regarding the application mode, tDCS seems to be effective in the rehabilitation of gait and balance, showing promising results in an anodic and dual application. Regarding cathodic application, its efficacy could not be determined due to the inclusion of only two studies of these characteristics. However, one of these studies stated that it is just as effective as anodic application. Khedr et al. [43], in a sample of 40 stroke patients, observed no differences between anodic or cathodic stimulation; both significantly improved function compared to sham-treated patients, but there were no significant differences between the two groups. The differences between Khedr et al. [43] and Fusco et al. [29] could be explained by the use of lower intensities than recommended and the small sample size in the Fusco study. The study by Andrade et al. [27] compared different application modes (anodic, cathodic, dual, or sham) and concluded that all groups showed a significant improvement compared to the sham group and the group that received the dual-tDCS stimulation showed significant improvements in respect of all types of stimulation in terms of gait and balance. Future research will be necessary to affirm which type of current is most effective in the rehabilitation of parameters related to the lower limbs; dual stimulation (anode in ipsilateral hemisphere to stroke, cathode in the contralateral hemisphere) appears to be a promising treatment option.

Regarding the stroke stage, in the acute and sub-acute stage, Tahatis et al. [35], Sohn et al. [34], and Andrade et al. [27] found significant improvements in balance; Chang et al. [28] found significant improvements in the functionality of the lower limb, and Andrade et al. [27] found improvements in gait and the RF. However, Leon et al. [44] found no significant improvement in the study of 49 subjects with subacute stroke in which they studied the application of tDCS combined with RAGt. No further studies were found evaluating gait and balance in these stages of stroke, so they were compared with articles that studied the application of tDCS in the upper limb. In the study carried out by Di Lazzaro et al. [45], significant changes were found in the inter-hemispheric imbalance in acute stroke subjects when they studied the use of the tDCS combined with constraint-induced movement therapy measured by evoked motor potentials. However, they did not find significant differences in the user manual function clinical scale. The included studies that did not find significant improvements were those that worked with intensities lower than recommended [29,33], so tDCS seems to be effective in the rehabilitation of the lower limbs in patients in acute and subacute stages. Klomjai et al.’s [30] study did not show significant improvements, although the parameters used were those recommended.

Regarding chronic stroke patients, Park et al. [32] and Ojardias et al. [31] found significant improvements in gait parameters. These results are consistent with the changes found by Danzl et al. [39] in the cortical excitability measured by evoked motor potentials and Seo et al. [41] in terms of gait and functionality in chronic stroke patients treated by RAGt and tDCS. There were two studies that did not show significant improvements [29,36]. Differences could be the application time since Utarapichat et al. [36] applied only 10 min of stimulation compared to 15 and 20 that applied the studies that found significant improvements, and intensities since Fusco et al. [29] applied a lower intensity than recommended. Thus, tDCS seems to be an effective treatment in acute, sub-acute, and chronic stroke patients. 

For future research and clinical application in relation to stroke patients and the parameters related to the lower limb, such as gait, balance, functionality, or strength, it seems that there are improvements in all stages of the disease when applying tDCS and conventional PT. Several treatment sessions seem to achieve long-lasting effects on cortical excitability, being essential applications of 2 mA on the region of the leg of the primary motor area to achieve excitability brain changes. Dual tDCS appears to be a promising treatment option, although it needs further study.

### Study Limitations

There were limitations related to the methodology and the low number of studies that compared the use of tDCS together with conventional PT. Regarding the methodology, only articles in English or Spanish were chosen, without limiting the methodological quality (three studies presented a methodological quality classified as “poor”), so the results of the studies have to be read carefully. Three studies were included which did not report whether their subjects performed any type of PT; these were included because they met the rest of the criteria and used only tDCS and no other intervention.

The included studies showed differences in the number of sessions, ranging from 1 to 16, but the cases that performed interventions of more than one session were homogeneous in their frequency (between 4 days/week and 5 days/week). A small number of studies were found to carry out the discussion. Because of this, we included a study on a pathology other than stroke and a study that carried out an intervention other than gait, balance, or lower limb training.

## 5. Conclusions

The use of tDCS combined with PT improves gait parameters, static and dynamic balance, and lower limb functionality in stroke patients. The parameters that have shown improvements are 2 mA for at least 10 min, with anodic or bihemispheric stimulation. These parameters have shown improvements at any stage of stroke in single or multiple session protocols, but their long-term efficacy has not been demonstrated. A greater number of comparative studies of modalities will be necessary to determine which method is more effective.

## Figures and Tables

**Table 1:** Medline Complete search strategy.

N°	Terms
1	Stroke OR cerebrovascular accident OR CVA ^1^.
2	Transcranial direct current stimulation OR tDCS ^2^.
3	Gait OR gait disorders, neurologic OR gait training OR neurological gait OR gait parameters OR balance OR stability OR postural balance OR postural stability OR kinematic OR kinetic OR gait analysis
4	Physiotherapy OR physical therapy OR rehabilitation OR exercise OR intervention
5	1 AND 2 AND 3 AND 3

^1^ CVA, cerebrovascular accident; ^2^ tDCS, transcranial direct current stimulation.

**Table 2:** Main results of the studies.

Trial	N	Assessment Tool	Intervention	Protocol	Results
Andrade et al., 2011	60, Acute	RF, BBS, 6MWT	tDCS + PT. A: anodal tDCS. B: bilateral tDCS. C: cathodal tDCS. D: sham tDCS. PT: 1 h/day, 3/week, in different times than tDCS	10 sessions, 2 weeks. Stimulation time not indicated. Multimodal tDCS, 2 mA, 35 cm^2^ electrodes. Anode: over the ipsilateral hemisphere to the stroke. Cathode: over the contralateral hemisphere to the stroke.	Baseline vs. post-treatment and 1 and 3 months follow-up. RF improved compared to sham (*p* < 0.05) after treatment and 3 months follow-up. No differences between real groups. BBS and 6MWT, significantly improved in real than in sham groups. Group B: more improvements in BBS (*p* = 0.001–*p* = 0.007), and in 6MWT (no significant) than A–C, respectively. Group A–C showed similar performance in all test (*p* > 0.005)
Chang et al., 2015	24, Acute	FAC, FMA-LE, BBS, TSGP	tDCS + PT. tDCS + PT. Sham tDCS + PT	10 sessions, 2 weeks. Anodal tDCS, 10 min, 2 mA, A: 7.07 cm^2^, C: 28.2 cm^2^. Anode over the tibialis anterior area of the ipsilateral hemisphere to the stroke.	Baseline vs. post-treatment. All motor functions improved significantly. FMA-LE improved significantly (*p* = 0.023) in tDCS group. No significant differences between groups in FAC (*p* = 0.077), BBS (0.759), and gait analysis.
Fusco et al., 2014	11, acute, sub-acute, chronic	TUG, 6MWT, 10MWT, RMI, FAC.	tDCS followed by PT. tDCS + PT. Sham tDCS + PT. PT: 2 days/week, 45 min.	10 sessions, 2 weeks. Cathodal tDCS, 10 min, 1.5 mA, 35 cm^2^ electrodes. Cathode: over the contralateral hemisphere to the stroke.	T0 baseline, T1 post-treatment, T2 1 month follow-up, T3 75–100 days after treatment. 10 MWT, 6MWT y TUG improved significantly in both groups after all measurements without differences between groups. FAC (*p* = 0.931) RMI (*p* = 0.537) did not show significant changes between both groups
Klomjai et al., 2018	19, sub-acute	SKE, TUG.	tDCS followed by PT. Group 1: real tDCS followed by sham tDCS. Group 2: sham tDCS followed real tDCS. PT: 1 h.	1 session, 1 sham session, 1-week interval. Dual tDCS, 2 mA, 20 min, 35 cm^2^ electrodes. Electrodes over the leg area of M1. Anode: over the ipsilateral hemisphere to the stroke. Cathode: over the contralateral hemisphere to the stroke.	Baseline vs. post-treatment. TUG increased significantly in both groups, but both groups did not differ No change in strength was found in either both groups
Ojardias et al., 2019	18, chronic	6MWT, TSGP, CDP	tDCS compared to sham. Group 1: real tDCS followed by sham tDCS. Group 2: sham tDCS followed real tDCS. No information about PT.	1session, 1 sham session, 11 days interval. Anodal tDCS, 2 mA, 20 min, 25 cm^2^ electrodes. Anode: over the ipsilateral hemisphere to the stroke. Leg area of M1.	T0 baseline, T1 9 days after first treatment, T2 9 days after second treatment. 6MWT significantly improved on real tDCS after 1 h after stimulation (*p* = 0.038). No significant differences were observed for the other evaluation.
Park et al., 2015	24, chronic	TSGP.	tDCS + TRT PT. TRT group. TRT + sham tDCS (TST) TRT + tDCS (TT) 30 min of TRT.	12 sessions, 3 days/week, 4 weeks Dual tDCS, 2 mA, 15 min. Electrodes over the leg area of M1. Anode: over the ipsilateral hemisphere to the stroke. Cathode: over the contralateral hemisphere to the stroke.	Baseline vs. post-treatment. All groups showed improvements in SPSP, SWPSP, and gait velocity. TT and TRT showed significant improvements (*p* < 0.05). TT group significantly improved compared with the TRT group
Saeys et al., 2015	31, acute, sub-acute	POMA. RMA.	tDCS + PT + TO. Group 1: real tDCS followed by sham tDCS. Group 2: sham tDCS followed real tDCS. PT + TO: 2 h, 5 times/week.	16 sessions/4 weeks, 16 sham sessions/4 weeks. Dual tDCS, 1.5 mA, 15 min, 35 cm^2^ electrodes. Electrodes over the motor cortex (C3-C4 areas). Anode: over the ipsilateral hemisphere to the stroke. Cathode: over the contralateral hemisphere to the stroke.	T1 baseline, T2 mid of study, T3 post-treatment. Both groups improved significantly on all outcome measures (*p* < 0.001) without differences between both (*p* > 0.005). POMA significantly improved in real group at middle of the study (4 weeks) (*p* = 0.049). RMA showed an improvement on the leg and trunk sub-score (*p* = 0.045) in real group.
Sohn et al., 2013	11, acute	SB, SKE	tDCS + PT. Group 1: real tDCS followed by sham tDCS. Group 2: sham tDCS followed real tDCS. No information PT frequency.	1 session, 1 sham session, 48 h interval. Anodal tDCS, 2 mA, 10 min, 35 cm^2^ electrodes. Anode: over the ipsilateral hemisphere to the stroke	Baseline vs. post-treatment. SB. Significant improvements with eyes opened and closed after real tDCS (*p* < 0.05). SKE. Significant improvements after real tDCS (*p* < 0.05)
Tahtis et al., 2014	14, sub-acute	TUG, POMA	tDCS compared to sham. Real tDCS. Sham tDCS. No information about PT.	1 session. Dual tDCS, 2 mA, 15 min, 35 cm^2^ electrodes. Electrodes over the leg area of M1. Anode: over the ipsilateral hemisphere to the stroke. Cathode: over the contralateral hemisphere to the stroke.	Baseline vs. post-treatment. TUG improved significantly in real than sham group (*p* = 0.018). POMA showed no differences between groups (*p* = 0.897).
Utarapichat et al., 2018	10, chronic	RMS and MF of VMO and TA, TUG.	tDCS compared to sham. Group 1: real tDCS followed by sham tDCS. Group 2: sham tDCS followed real tDCS. PT frequency not indicated.	1 session, 1 sham session, 48 h interval. Anodal tDCS, 2 mA, 10 min, A: 10.1 cm^2^, C: 25 cm^2^. Anode over the leg area of M1 (ipsilateral hemisphere to the stroke).	Baseline vs. post-treatment. There were not differences between tDCS and sham stimulation (*p* > 0.05) all measures.

6MWT, 6-min walking test; 10MWT, 10-m walking test; A, anode; BBS, Berg Balance Scale; C, cathode; CDP; Computerized dynamic posturography, FMA-LE, Fugl–Meyer Assessment lower limb, FAC, Functional ambulatory Category; Fugl-Meyer Assessment lower limb sub-scale; M1, primary motor cortex; MF, median frequency; PT, physiotherapy; POMA, Tinetti Performance Oriented Mobility Assessment; RF, rate of falls; RMI, Rivermead Mobility Index; RMA, Rivermead Motor Assessment; RMS, root mean square; SPSP, stance phase symmetry profile; SB, static balance; SKE, strength of knee extensor; SWPSP, swing phase symmetry profile; TRT, task related training; TSGP, temporospatial gait parameters; TA, tibialis anterior, TUG, timed-up and go; VMO, vastus medialis oblique.

## Data Availability

Not applicable.

## Appendix A

Articles excluded in the systematic review:

- Danzl MM, Chelette KC, Lee K, Lykins D, Sawaki L. Brain stimulation paired with novel locomotor training with robotic gait orthosis in chronic stroke: a feasibility study. NeuroRehabilitation, 2013;33(1):67-76. Article excluded because it uses robotic therapy
- Geroin C, Picelli A, Munari D, Waldner A, Tomelleri C, Smania N. Combined transcranial direct current stimulation and robot-assisted gait training in patients with chronic stroke: a preliminary comparison. Clin Rehabi, 25(6):537–48. Article excluded because it uses robotic therapy
- Leon D, Cortes M, Elder J, Kumru H, Laxe S, Edwards DJ, et al. tDCS does not enhance the effects of robot-assisted gait training in patients with subacute stroke. Restor Neurol Neurosci, 2017;35(4):377–84. Article excluded because it uses robotic therapy
- Seo HG, Lee WH, Lee SH, Yi Y, Kim KD, Oh B-M. Robotic-assisted gait training combined with transcranial direct current stimulation in chronic stroke patients: A pilot double-blind, randomized controlled trial. Restor Neurol Neurosci, 2017;35(5):527–36. Article excluded because it uses robotic therapy
- Picelli A, Chemello E, Castellazzi P, Roncari L, Waldner A, Saltuari L, et al. Combined effects of transcranial direct current stimulation (tDCS) and transcutaneous spinal direct current stimulation (tsDCS) on robot-assisted gait training in patients with chronic stroke: A pilot, double blind, randomized controlled trial. Restor Neurol Neurosci, 2015;33(3):357–68. Article excluded because it uses other stimulations (spinal direct current stimulation)
- Picelli A, Brugnera A, Filippetti M, Mattiuz N, Chemello E, Modenese A, et al. Effects of two different protocols of cerebellar transcranial direct current stimulation combined with transcutaneous spinal direct current stimulation on robot-assisted gait training in patients with chronic supratentorial stroke: A single blind, randomized controlled trial. Restor Neurol Neurosci. 2019;37(2):97–107. Article excluded because it uses other stimulations (cerebellar transcranial direct current stimulation)
- Picelli A, Chemello E, Castellazzi P, Filippetti M, Brugnera A, Gandolfi M, et al. Combined effects of cerebellar transcranial direct current stimulation and transcutaneous spinal direct current stimulation on robot-assisted gait training in patients with chronic brain stroke: A pilot, single blind, randomized controlled trial. Restor Neurol Neurosci, 2018;36(2):161–71. Article excluded because it uses other stimulations (cerebellar transcranial direct current stimulation)
- Ang KK, Guan C, Phua KS, Wang C, Zhao L, Teo WP, et al. Facilitating effects of transcranial direct current stimulation on motor imagery brain-computer interface with robotic feedback for stroke rehabilitation. Arch Phys Med Rehabil, 2015;96(3):S79–87. Article excluded because it uses robotic therapy
- Cho HS, Cha HG. Effect of mirror therapy with tDCS on functional recovery of the upper extremity of stroke patients. Journal of physical therapy science, 2015;27(4), 1045-47. Article excluded because it assesses the upper limb
- Zheng X, Schlaug G. Structural white matter changes in descending motor tracts correlate with improvements in motor impairment after undergoing a treatment course of tDCS and physical therapy. Frontiers in human neuroscience, 2015;9, 229. Article excluded because it assesses the upper limb
- Allman C, Amadi U, Winkler AM, Wilkins L, Filippini N, et al. Ipsilesional anodal tDCS enhances the functional benefits of rehabilitation in patients after stroke. Science translational medicine, 2016;8(330),330re1-330re1. Article excluded because it assesses the upper limb
- Rocha S, Silva E, Foerster Á, Wiesiolek C, Chagas AP, et al. The impact of transcranial direct current stimulation (tDCS) combined with modified constraint-induced movement therapy (mCIMT) on upper limb function in chronic stroke: a double-blind randomized controlled trial. Disability and rehabilitation, 2016;38(7), 653-60. Article excluded because it assesses the modified constraint-induced movement therapy
- Cha HK, Ji SG, Kim MK, Chang JS. Effect of transcranial direct current stimulation of function in patients with stroke. Journal of physical therapy science, 2014;26(3), 363-65. Article excluded because it assesses the upper limb
- Tedesco Triccas L, Burridge JH, Hughes AM, Meadmore KL, Donovan-Hall M, Rothwell JC, et al. A qualitative study exploring views and experiences of people with stroke undergoing transcranial direct current stimulation and upper limb robot therapy. Top Stroke Rehabil, 2018;20,1–9. Article excluded because it uses a qualitative design
- Sattler V, Acket B, Raposo N, Albucher JF, Thalamas C, et al. Anodal tDCS combined with radial nerve stimulation promotes hand motor recovery in the acute phase after ischemic stroke. Neurorehabilitation and neural repair, 2015;29(8), 743-54. Article excluded because it uses other stimulations (radial nerve stimulation)
- Ilić NV, Dubljanin-Raspopović E, Nedeljković U, Tomanović-Vujadinović S, Milanović SD, et al. Effects of anodal tDCS and occupational therapy on fine motor skill deficits in patients with chronic stroke. Restorative neurology and neuroscience, 2016;34(6), 935-945. Article excluded because it assesses the upper limb
- Bolognini N, Vallar G, Casati C, Latif LA, El-Nazer R, Williams, et al. Neurophysiological and behavioral effects of tDCS combined with constraint-induced movement therapy in poststroke patients. Neurorehabilitation and neural repair, 2011;25(9), 819-29. Article excluded because it assesses the modified constraint-induced movement therapy
- Flöel A. tDCS-enhanced motor and cognitive function in neurological diseases. Neuroimage, 2014;85, 934-47. Article excluded because it is a systematic review
- Schlaug G, Renga V, Nair D. Transcranial direct current stimulation in stroke recovery. Archives of neurology, 2008;65(12), 1571-1576. Article excluded because it is a systematic review
- Lefaucheur JP, Antal A, Ayache SS, Benninger DH, Brunelin J, Cogiamanian, et al. Evidence-based guidelines on the therapeutic use of transcranial direct current stimulation (tDCS). Clinical Neurophysiology, 2017;128(1),56-92. Article excluded because it is a systematic review
- Ambrosini E, Ferrante S, Ferrigno G, Molteni F, Pedrocchi A. Cycling induced by electrical stimulation improves muscle activation and symmetry during pedaling in hemiparetic patients. IEEE Trans neural Syst Rehabil Eng a Publ IEEE Eng Med Biol Soc. 2012;20(3):320–30. Article excluded because it uses other stimulations
- Yotnuengnit P, Bhidayasiri R, Donkhan R, Chaluaysrimuang J, Piravej K. Effects of transcranial direct current stimulation plus physical therapy on gait in patients with Parkinson disease: a randomized controlled trial. American journal of physical medicine & rehabilitation, 2018;97(1), 7-15. Article excluded because it included participants with Parkinson disease
- Grecco LA, Duarte NA, Zanon N, Galli M, Fregni F, Oliveira CS. Effect of a single session of transcranial direct-current stimulation on balance and spatiotemporal gait variables in children with cerebral palsy: a randomized sham-controlled study. Brazilian journal of physical therapy, 2014;18(5), 419-27. Article excluded because it included participants with cerebral palsy
- Kumru H, Murillo N, Benito-Penalva J, Tormos JM, Vidal J. Transcranial direct current stimulation is not effective in the motor strength and gait recovery following motor incomplete spinal cord injury during Lokomat^®^ gait training. Neuroscience letters, 2016;620,143-47. Article excluded because it included participants with Spinal Cord Injury
- Kaski D, Dominguez RO, Allum JH, Islam AF, Bronstein AM. Combining physical training with transcranial direct current stimulation to improve gait in Parkinson’s disease: a pilot randomized controlled study. Clinical rehabilitation, 2014;28(11),1115-24. Article excluded because it included participants with Parkinson disease
